# Breast Implant-Associated Anaplastic Large Cell Lymphoma: A Case Report About a Patient with Cytology Negative for Malignancy

**DOI:** 10.3390/life14111494

**Published:** 2024-11-16

**Authors:** Didem Ün, Johannes Rohrbeck, Mathias Drach, Robert Ullrich, Philipp B. Staber, Thomas H. Helbich, Christian Freystätter, Maryana Teufelsbauer, Christine Radtke

**Affiliations:** 1 Department of Plastic, Reconstructive and Aesthetic Surgery, Medical University of Vienna, Spitalgasse 23, 1090 Vienna, Austria; didem.uen@meduniwien.ac.at (D.Ü.); christian.freystaetter@meduniwien.ac.at (C.F.); maryana.teufelsbauer@meduniwien.ac.at (M.T.); 2 Institute of Pharmacology, Center for Physiology and Pharmacology, Medical University of Vienna, Währinger Straße 13a, 1090 Vienna, Austria; 3 Department of Pathology, Medical University of Vienna, Spitalgasse 23, 1090 Vienna, Austria; johannes.rohrbeck@meduniwien.ac.at (J.R.); robert.ullrich@meduniwien.ac.at (R.U.); 4 Department of Dermatology, Medical University of Vienna, Spitalgasse 23, 1090 Vienna, Austria; mathias.drach@meduniwien.ac.at; 5 Division of Hematology and Hemostaseology, Department of Internal Medicine I, Medical University of Vienna, Spitalgasse 23, 1090 Vienna, Austria; philipp.staber@meduniwien.ac.at; 6 Division of General and Pediatric Radiology, Department of Biomedical Imaging and Image-Guided Therapy, Medical University of Vienna, Spitalgasse 23, 1090 Vienna, Austria; thomas.helbich@meduniwien.ac.at

**Keywords:** breast cancer, breast implants, breast implant complications, breast surgery, breast imaging

## Abstract

Breast implant-associated anaplastic large cell lymphoma (BIA-ALCL) is a rare lymphoma primarily linked to textured breast implants. Symptoms are often non-specific (e.g., breast swelling, pain, or fluid collection). When imaging detects fluid around the implant, cytological examination is the first diagnostic approach. However, this method has limited sensitivity and may yield false-negative results. In this case, a 41-year-old woman presented with swelling, pain, and itching in her left breast six years after bilateral textured breast implant placement. Ultrasonography (US) revealed peri-implant fluid collection around the left implant. A following magnetic resonance imaging (MRI) scan ruled out an implant rupture. Due to persistent pain and the peri-implant effusion on the left side, open surgery was performed. During implant removal, the seroma was drained, and multiple suspicious masses were found on the left side. The cytology of the seroma fluid was negative and intraoperative frozen sections of the excised masses were inconclusive. A complete capsulectomy was conducted due to the suspicion of malignancy. Histological examination ultimately confirmed the diagnosis of BIA-ALCL. This case highlights the diagnostic challenges associated with this rare condition. Therefore, BIA-ALCL should always be considered in the differential diagnosis of breast implant-associated seroma.

## 1. Introduction

Breast implant-associated anaplastic large cell lymphoma (BIA-ALCL) is a rare type of T-cell non-Hodgkin lymphoma primarily associated with textured breast implants. First reported in 1997 by Keech and Creech, more than 1570 cases have been identified worldwide to date [1,2]. The etiology of BIA-ALCL remains uncertain, with the current hypothesis considering the combination of chronic inflammation and genetic factors (e.g., TP53 and BRCA 1/2 germline mutations) [3]. Patients commonly present with spontaneous peri-prosthetic effusion, breast enlargement, swelling, and pain. Less frequently, it can manifest as a capsule contracture, tumor mass, axillary lymphadenopathy, skin rash or as systemic symptoms [4,5]. In cases of suspected BIA-ALCL, ultrasonography (US) should be the initial diagnostic step. If US detects peri-prosthetic fluid, US-guided fine needle aspiration (FNA) and pathological examination are advised. In cases of suspected implant rupture, magnetic resonance imaging (MRI) should be performed for further evaluation [6]. The diagnosis of BIA-ALCL is confirmed by its characteristic morphology and immunophenotype being CD30 positive and anaplastic lymphoma kinase (ALK) negative [5,7]. The primary treatment involves surgical intervention, including implant removal, en-bloc capsulectomy, and the excision of any tumor masses if present. Depending on the extent of disease, patients receive adjuvant chemotherapy, immunotherapy and/or radiotherapy. Follow-up examinations include contrast-enhanced computed tomography (CT) or positron emission tomography (PET)-CT scans [4,5].

## 2. Case Report

**Figure 1:**
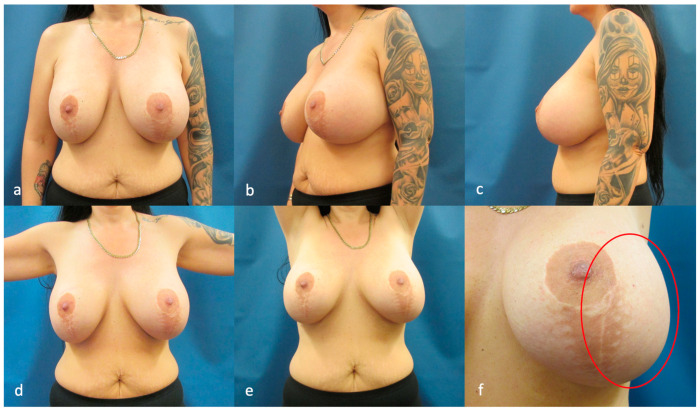
Preoperative views show breast asymmetry. (**a**) Frontal view. (**b**) Oblique view. (**c**) Lateral view of left breast. (**d**) Frontal view with arms abducted. (**e**) Frontal view with arms raised over the head. (**f**) Close-up view of the left breast, with apparent enlargement relative to the right breast (indicated by a red circle).

A 41-year-old white female patient presented with a 6-week history of spontaneous unilateral breast swelling, pain, and an intramammary itching of her left breast. She had undergone bilateral breast augmentation using textured round high profile gel breast implants with a volume of 535cc six years ago in Hungary. She reported smoking three cigarettes daily. The patient has a positive family history for breast cancer on her maternal side. Her cup size was 80G and her BMI was 27.3. On physical examination, her left breast was noted to be swollen in comparison to the right (Figure 1).

**Figure 2:**
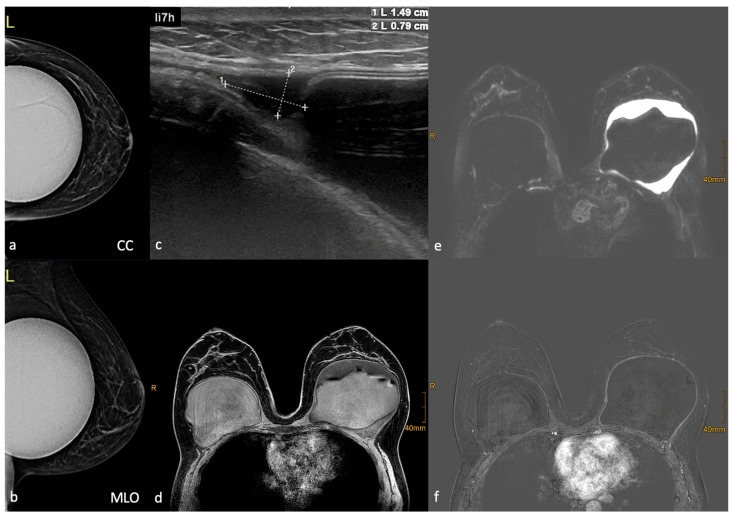
Imaging studies of the breast. (**a**,**b**) Two view 3D mammography (tomosynthesis) of the left breast shows isolated benign calcifications and an implant in situ. (**c**) Sonography of the left breast shows a homogeneous hypoechoic fluid collection around the subpectoral implant. (**d**) Axial T1 weighted MRI without contrast material showing bilateral implants. The left side shows fluid accumulation within the implant capsule and wrinkling of the implant. (**e**) Axial T2 fat-saturated MRI shows a high-signal fluid accumulation around the left implant, while the right implant remains unremarkable. (**f**) Axial T1 weighted MRI image with contrast material indicates capsule enhancement.

The patient initially underwent 3D mammography (tomosynthesis). The examination provided no evidence of malignant changes and showed dense implants (Figure 2a,b). A subsequent US revealed fluid surrounding the implant on the left side (Figure 2c). An MRI of the breast was performed to rule out implant rupture. This showed a peri-implant effusion on the left side, but no evidence of rupture (Figure 2d,e). An enhancement of the fibrous capsule and a lymphadenopathy in the left axilla were noted (Figure 2f). Surgery was indicated due to the patient’s persistent pain and the peri-implant effusion on the left side.

**Figure 3:**
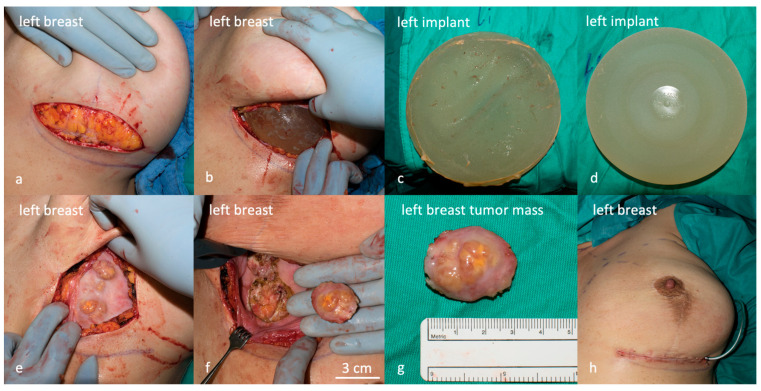
Gross anatomical representation of the affected area during surgery. (**a**) The incision was made along the existing scar. (**b**) The implant was exposed. (**c**) The anterior surface of the textured breast implant with adhered fibrous tissue is shown. (**d**) The posterior surface depicts the labeling of the intact implant. (**e**) After the removal of the implants, the masses within the capsule were noticed. (**f**) The masses were removed by resection. (**g**) The tumors had a size between 1 and 3 cm. (**h**) Immediate cosmetic results after bilateral implant removal and capsulectomy are shown.

**Figure 4:**
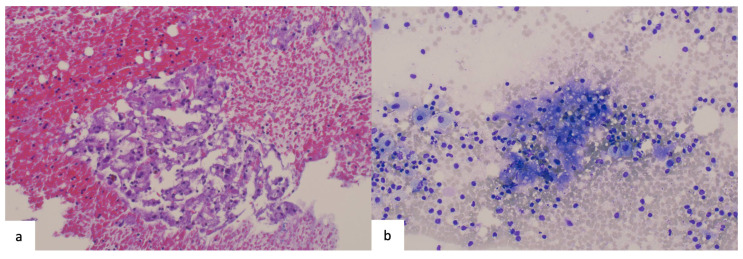
The cytological results are negative for malignancy. (**a**) The cell block shows a foreign body reaction accompanied by foam cells (hematoxylin and eosin stain; magnification: 100×). (**b**) The section does not exhibit BIA-ALCL-typical blasts (Giemsa stain; magnification: 100×).

**Figure 5:**
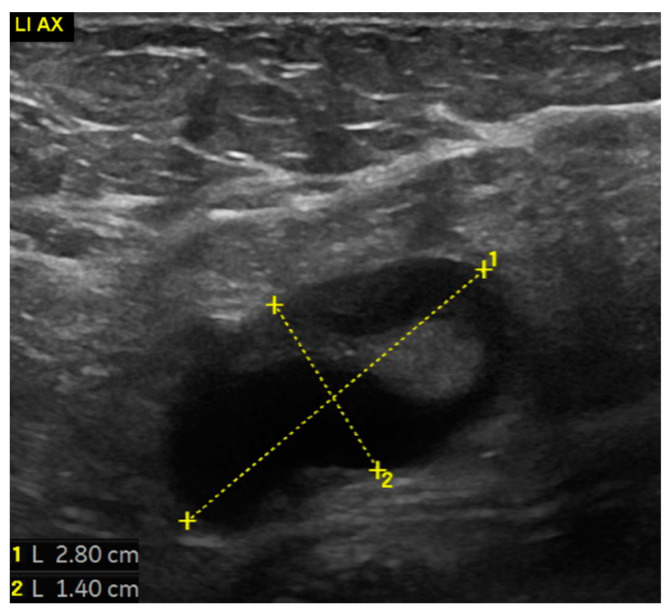
Ultrasonography (US) of the left axilla indicates lymphadenopathy. The lymph node measures 2.8 cm at its longest axis and 1.4 cm at its shortest axis, consistent with enlargement.

A submammary skin incision was made and the implants were exposed (Figure 3a,b). During this step, the seroma was drained from the left side and sent for cytological examination. The analysis showed foam cells and a foreign body reaction, with no evidence of malignancy (Figure 4). The implants were removed and were intact (Figure 3c,d). The fibrous tissue adhered to the implant surface was sent for culture, which was found to be sterile. Several suspicious masses were found and excised from within the capsule on the left side (Figure 3e–g). These masses were then used to prepare intraoperative frozen sections, but the results were inconclusive. Subsequently, a complete capsulectomy was performed as there was a suspicion of malignancy. The tissue was then sent for histological examination together with the former breast augmentation scars. The right side was inconspicuous and free of tumors. Both breast cavities were washed and closed with a drain without inserting new implants (Figure 3h). A postoperative US revealed pathologically suspicious lymph nodes in the left axilla (Figure 5).

**Figure 6:**
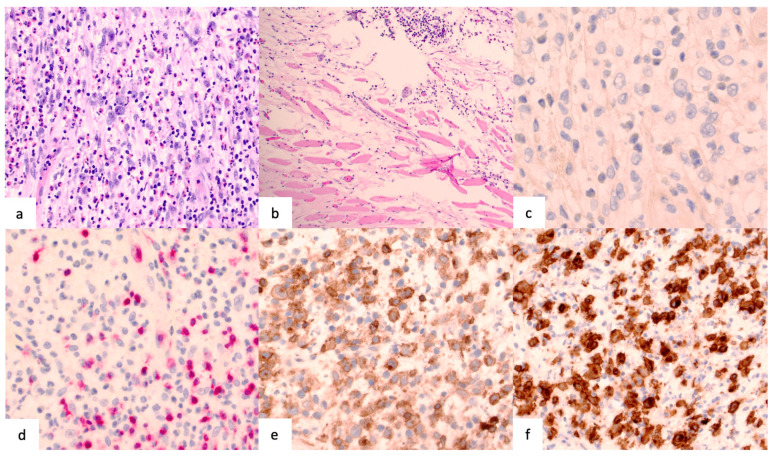
Histological and immunohistochemical features of breast implant-associated anaplastic large cell lymphoma (BIA-ALCL). (**a**) Section illustrating large, pleomorphic cells with occasional horseshoe-shaped nuclei (hematoxylin and eosin stain; magnification: 400×). (**b**) Lymphoma cells adjacent to the pectoralis major muscle, illustrating their close proximity (hematoxylin and eosin stain; magnification: 100×). (**c**) The cells are negative for anaplastic lymphoma kinase (ALK) (magnification: 400×). (**d**) The cells are negative for CD3 (magnification: 200×). (**e**) The blasts are positive for CD4 (magnification: 200×). (**f**) The cells are strongly positive for CD30 (magnification: 200×).

Histological examination revealed a capsule with multiple nodular distensions (Figure 6a). Within these nodules, sparsely arranged blastic cells are organized in aggregates, characterized by oval, kidney-shaped nuclei. The background includes small, chromatin-dense lymphocytes, individual plasma cells, histiocytes, and eosinophilic granulocytes. In some areas, the infiltration extends to within fractions of a millimeter from the muscle (Figure 6b). The cells are uniformly negative for ALK and CD3 but positive for CD4 and CD30 (Figure 6c–f). A BIA-ALCL diagnosis was confirmed based on these findings.

Immuno-chemotherapy started three weeks after diagnosis. The patient received brentuximab vedotin in combination with cyclophosphamide, hydroxydaunorubicin, and prednisolone (CHP) on a 21-day cycle for six cycles. An FDG-PET-CT scan was conducted to assess the effectiveness of the therapy. Multidisciplinary follow-up care was provided.

## 3. Discussion

We report on a patient that diverges from the typical diagnostic pattern of BIA-ALCL. The literature advocates for preoperative FNA and the thorough cytological examination of implant-associated seromas, which are often pathognomonic for BIA-ALCL [5,6]. In this case, open surgery was performed immediately due to the patient’s pain and the peri-implant effusion on the left side. Intraoperative samples were collected for cytological analysis, which showed no signs of malignancy. Only the histological workup of the intraoperatively removed tumor foci resulted in the diagnosis of BIA-ALCL. This case highlights the limitations of FNA in certain instances, suggesting that a negative cytological result alone does not definitively rule out BIA-ALCL. In the presence of an effusion, FNA should be performed, ensuring that at least 50 mL of fluid is collected for cytological analysis [6]. However, even if the cytological results are negative, further diagnostic evaluation including surgical intervention and histopathological examination should be pursued when clinical suspicion for BIA-ALCL remains high.

It should be taken into consideration that our patient has a positive family history of breast cancer. In patients with a genetic predisposition to breast cancer, particularly those with TP53 and BRCA1/2 germline mutations, BIA-ALCL prevalence appears to be higher, and the time to onset seems to be shorter compared to the general population [8,9]. Our patient developed the disease 6 years after implant placement. This is earlier than the mean onset of BIA-ALCL occurring 8–10 years after implantation [10,11]. This earlier onset could be related to the patient’s positive family history.

The incidence of BIA-ALCL among women with breast prostheses has been estimated between 1 in 355 and 1 in 559 patients [10,12]. Given this low incidence, it is important to raise awareness among medical professionals and patients. In response, the U.S. Food and Drug Administration (FDA) issued labeling guidelines with a warning in 2020: All women receiving implants, whether smooth or textured, are cautioned about the potential risk of BIA-ALCL, with the risk increasing in correlation to the roughness of texturing [13,14,15,16,17]. Furthermore, these low incidence rates pose a challenge in gathering enough data to establish clear correlations and detect the disease effectively. To further address this, we propose the implementation of a dedicated BIA-ALCL registry in Austria. Given the success of the American Register PROFILE in tracking BIA-ALCL cases, establishing a similar initiative in Austria is crucial [2]. It would advance our understanding of BIA-ALCL and improve disease management through more robust data collection.

## 4. Conclusions

BIA-ALCL remains a diagnostic challenge due to its rarity and often vague clinical presentation. Therefore, BIA-ALCL should be considered in the differential diagnosis for all cases of breast implant-associated seroma. FNA is a useful tool for initial assessment and should be utilized in these cases. However, its limited sensitivity can result in false-negative results. Therefore, in cases with a persistently high index of suspicion for BIA-ALCL, surgical intervention and comprehensive histological workup should be considered. This report underscores the need for increased awareness among healthcare providers and patients to facilitate timely diagnosis and treatment.

## Data Availability

The data presented in this study are available on request from the corresponding author. The data are not publicly available due to privacy and ethical restrictions.
